# The effect of phosphorus on solidification behaviour of undercooled Al–70 wt.%Si alloys

**DOI:** 10.1038/s41598-020-75402-0

**Published:** 2020-10-26

**Authors:** Bo Dang, Zengyun Jian, Junfeng Xu

**Affiliations:** The Shaanxi Key Laboratory of Photoelectric Functional Materials and Devices, Xi’an Technological University, Xi’an, 710021 Shaanxi People’s Republic of China

**Keywords:** Structural materials, Mechanical properties, Metals and alloys

## Abstract

Effect of refining element phosphorus (P) on the morphology of the primary silicon in the Al–70 wt.%Si alloy was investigated via the electromagnetic levitation (EML) technique. The morphology and microstructure were analyzed by using high-speed video (HSV) and scanning electron microscopy (SEM). It was found that the morphology of primary silicon transformed from dendrites with several branches to blocky shape, and then to equiaxed grains in Al–70 wt.%Si and Al–70 wt.%Si–1.0 wt.%P alloys with increasing of undercooling. The nucleation number and nucleation rate increased exponentially with the increase of undercooling for both alloys. Finally, the growth velocity of primary silicon was discussed in combination with classical theory.

Hypereutectic Al–Si alloys with high silicon become an important material, because of their low thermal expansion coefficient and excellent wear resistance, etc., and these alloys have been widely applied in the field of electronic packaging^1–4^. However, the coarse primary silicon deteriorates the properties and limits the application of this alloy. Many researchers tried to refine the primary silicon and investigated the corresponding refining mechanism. Based on the solidification theory, the crystal-morphology is related to Jackson factor^5^. The Jackson factor *α* is the product of a crystallographic factor and the ratio of the melting entropy to the gas constant^5,6^. For metals and some organic compounds with *α* < 2, the growth morphology has the characteristics of non-faceted phase; most of the inorganic compounds with *α* > 5, the growth morphology has the characteristics of faceted phase; while few materials with 2 < *α* < 5, such as Bi, Sb, Ga, Ge, and Si, the growth morphology has the characteristics of either non-faceted or faceted phase. The morphology of crystal depends on the growth direction for the materials with 2 < *α* < 5. Under near-equilibrium solidification condition, the anisotropy of primary silicon leads to the difference of morphology^7^. The morphology of primary silicon may be long and platelike with faceted growth mode, spherical with non-faceted growth mode, and block with mixture of faceted and non-faceted growth modes by controlling the solidification conditions.

According to the report^8–11^, adding elements is a common way to refine primary silicon for hypereutectic Al–Si alloys. The P is an effective element which is commonly used in factories. Chen et al.^8^ reported that the morphology of primary silicon in Al–20 wt.%Si alloy was modified obviously with P addition, and the surface morphology of primary silicon particle with P addition was mellower compared with that without P addition. Gao et al.^9^ observed that the in-situ formed AlP nucleus exhibited attractive performance on primary silicon particles in A390 melt. Nie et al.^10^ found that Ti_5_P_3.16_ particles were an efficient refiner for hypereutectic Al–Si alloy, the average size of primary silicon decreased from 85 to 12.5 μm. Wang et al.^11^ found that the ultimate tensile strength and elongation of Al–30 wt.%Si alloy increased by approximately 13.7% and 136.6%, respectively, by the addition of the Al–P–O master alloy. Although there are many works about the P effect on the refinement of the primary Si in hypereutectic Al–Si alloys, the influence of P addition on the nucleation and growth of the primary silicon by dynamically observing in hypereutectic Al–Si alloys during solidification has not been reported yet. In this work, the effect of P addition on the morphologies of primary silicon for undercooled Al–70 wt.%Si and Al–70 wt.%Si–1.0 wt.%P alloys have been analyzed through observing the growth morphologies of primary silicon by high-speed video during solidification and measuring the nucleation number, growth velocities of primary silicon in a wide range of undercooling.

## Results

### Temperature curve

**Figure 1:**
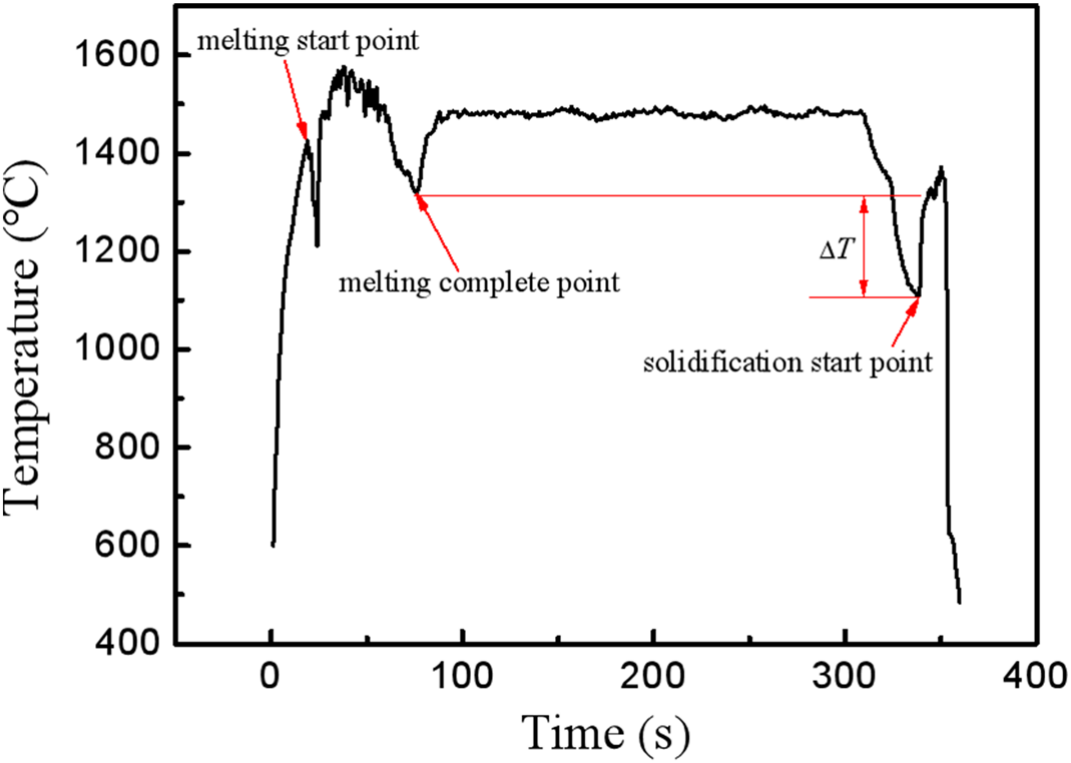
The typical temperature curve for the sample during melting and solidification.

Since the emissivity of solid silicon is much higher than that in liquid, the temperature measured by infrared thermometer for liquid and solid silicon at the same temperature must be different. Therefore, when the sample is in solid–liquid mixed state, the curve of temperature vs. time is a fluctuating curve due to its continuous rotation. If the heating power is constant, and the sample is melted completely, an obvious abrupt change will occur in the temperature curve. Therefore, the temperature of the alloy melt measured by the infrared thermometer can be determined from the temperature–time curve during the sample heating process. Figure 1 is the typical temperature curve for the sample during melting and solidification. According to the liquidus temperature and the temperature measured by the infrared thermometer, the emissivity of the alloy melt under the experimental conditions was calculated by Eq. (1)^12^. The material parameters and calculated results are listed in Table 1. The nucleation temperature and the corresponding undercooling of the sample can be obtained through the Eq. (1) based on the nucleation temperature measured from infrared thermometer and the emissivity of the alloy melt.

1 $$ M\left( {\lambda ,T} \right) = \frac{{C_{1} }}{{\lambda^{5} }}\left( {e^{{\frac{{C_{2} }}{\lambda T}}} - 1} \right)^{ - 1} $$

**Table 1:** The emissivity of Al–70 wt.%Si alloy.

Real melting (*T*_1_/K)^13^	Recorded melting (*T*_2_/K)	Wavelength of spectra radiation (λ/µm)	Real emissivity (ε_1_)	Set emissivity (ε_2_)
1516	1128	1.6	0.132	1

where *M*(*λ*, *T*) is the light radiant exitance, λ is wavelength (the λ = 1.6 μm in this experiment), *T* is thermodynamic temperature (K), *C*_1_ is the first radiation constant, and the *C*_1_ = 3.7418 × 10^−12^(W cm^2^), *C*_2_ is the second radiation constant, and the *C*_2_ = 1.4388(cm K).

### Growth morphologies

**Figure 2:**
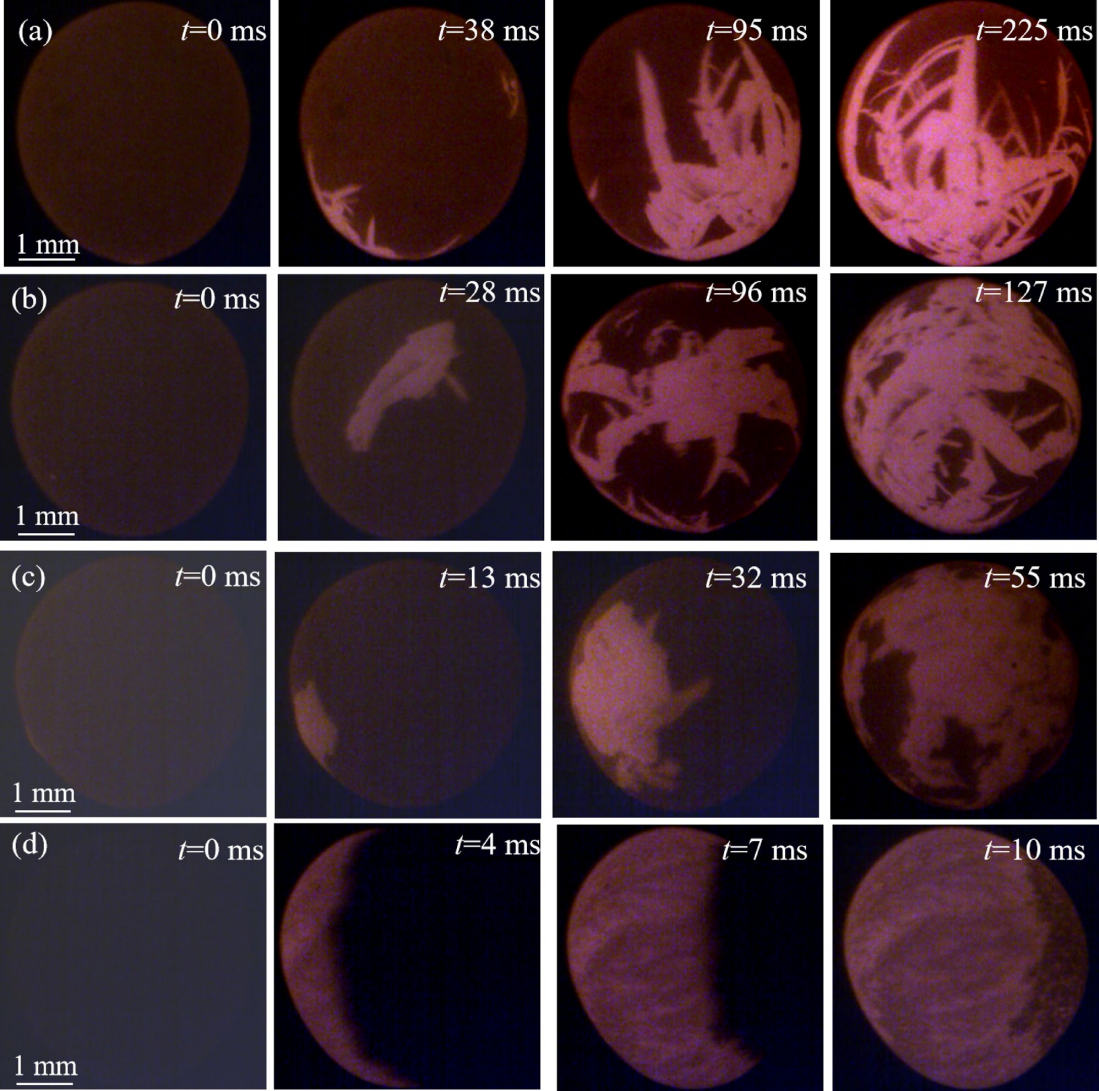
The HSV images of the Al–70 wt.%Si alloys at different undercooling: (**a**) Δ*T* = 92 K, (**b**) Δ*T* = 109 K, (**c**) Δ*T* = 154 K, (**d**) Δ*T* = 240 K.

Figure 2 shows the growth morphology of primary silicon during solidification for Al–70 wt.%Si alloys at different undercooling. The dark, gray and bright parts in HSV images are the undercooled liquid, the reheated liquid resulting from the release of latent heat, and the solid crystals, respectively. It can be seen that the solid–liquid interface is different from the crystal–liquid interface at different undercoling. *t* = 0 ms is the time of the primary silicon starts to solidify from the Al–Si melt that can be observed by HSV.

At low undercooling, the primary silicon grows from a small point to one or several long and thin plate-like shape with times going on at the surface of the undercooled melt, as shown in Fig. 2a,b. It should be pointed that when the undercooling is smaller, the primary silicon initially grows to be a thin plate in undercooled liquid. With the increase of undercooling, the sample’s interface appears as several silicon dendrites, as shown in Fig. 2b, and the morphology of primary silicon grows to be a plate with several branches. Meanwhile, the primary silicon has a higher growth rate in the radial direction of the plate and a lower growth rate in the direction perpendicular to the plate. The growth mode of primary silicon shows obvious anisotropy. In addition, only two color areas can be distinguished in the HSV images. The reason is that the brightness of undercooled liquid and the reheated liquid is very small at low undercooling, which can not be distinguished by the HSV.

Figure 2c shows the HSV images of the sample with Δ*T* = 154 K, where the interface appears more nucleation sites, and the primary silicon solidified as many blocky grains, instead of dendrite with branches. It is worth to note that the crystals cannot be detected during the recalescence process, and the recalescence interface presents a shape of the massif.

When the undercooling is higher than 240 K, as shown in Fig. 2d, the morphology of primary silicon shows as equiaxed grains, and the recalescence interface transforms into a parallel and smooth one.

**Figure 3:**
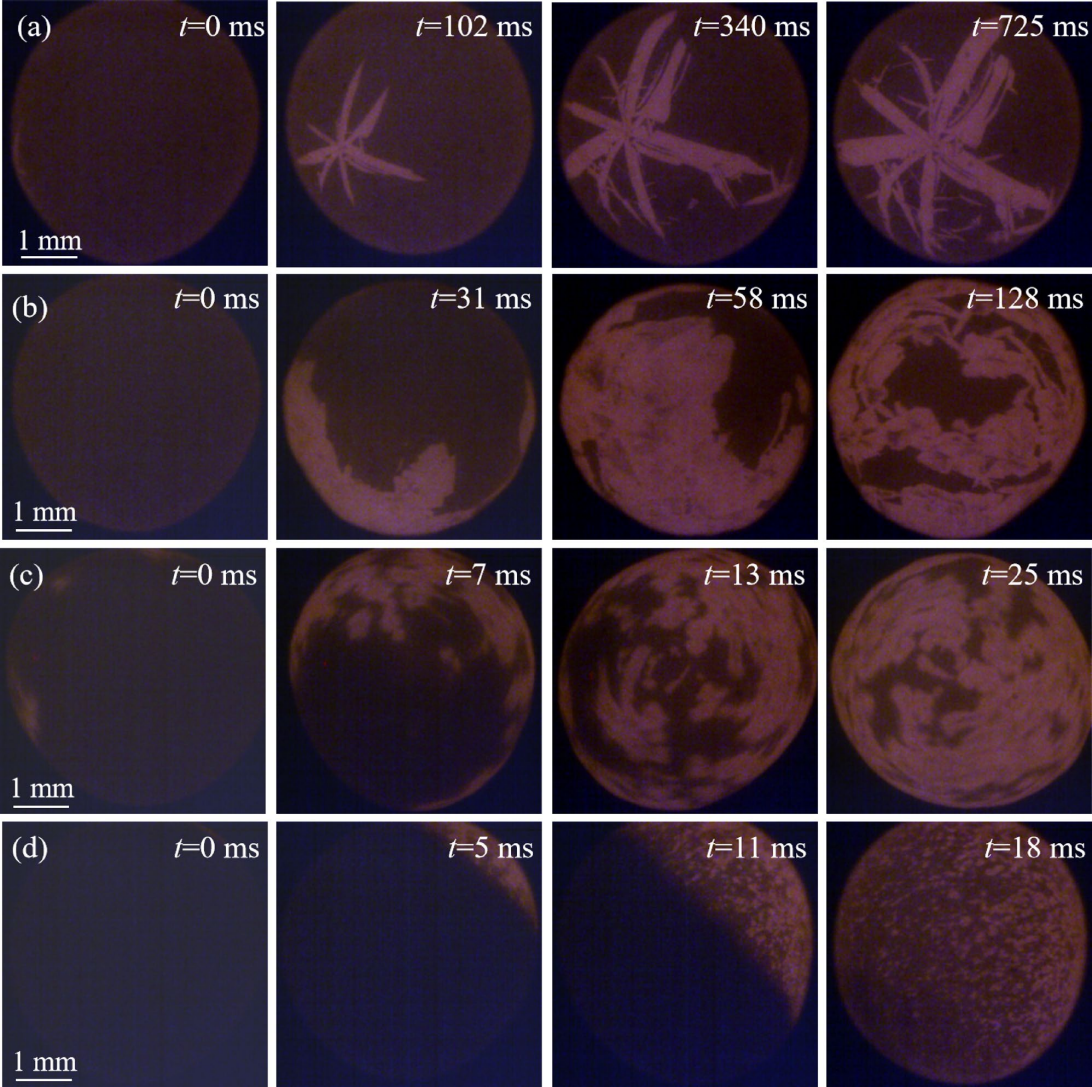
The HSV images of the Al–70 wt.%Si–1.0 wt.%P alloys at different undercooling: (**a**) Δ*T* = 51 K, (**b**) Δ*T* = 104 K, (**c**) Δ*T* = 141 K, (**d**) Δ*T* = 190 K.

Figure 3 shows the morphology of primary silicon for Al–70 wt.%Si–1.0 wt.%P alloy at different undercooling. When the undercooling is 51 K, the grains grow into dendrites with several branches. As time goes on, the newly formed dendrites at different sites moved into the solid phase region gradually. In Fig. 3b,c, the morphology of primary silicon grows in the shape of a fragmented and blocky shape, which confirms the tendency of solid phase agglomerating. When undercooling further increased to Δ*T* = 190 K, undercooling, the HSV images shows that almost all primary silicon is equiaxed grain. The solid–liquid interface (bright) and the heat interface (gray) that can be observed by HSV.

In summary, the morphology of primary silicon depends on the nucleation undercooling. With increasing of undercooling, the morphology of primary silicon transforms from dendrites with several branches to blocky shape, and then to equiaxed grains. The growing morphology in the solidification process is determined by the growth mode of the crystal^14^. The primary silicon is a dendrite at low undercooling, showing the feature of lateral growth. In intermediate undercooling, the morphology of primary silicon not only shows some feature of lateral growth, but also shows some feature of non-lateral growth. It can be speculated that the primary silicon grows in a mixed growth mode. When the undercooling is large enough, the primary silicon has no anisotropy in the growth direction. It can be concluded that the primary silicon grows in a non-lateral growth mode.

The undercooling also affects the morphology of the recalescence interface. At low undercooling, the recalescence heat that from the release of latent heat is not sufficiently large, the recalescence interface can not be distinguished by the HSV. The morphology of the recalescence interface changed from rough to smooth with the undercooling increased from intermediate to high region.

According to the literature, the P was added into Al–Si alloy, formed AlP particles which has very similar crystal lattice with primary silicon^4,15^. They can act as heterogeneous nuclei for the primary silicon and promote the nucleation rate of the primary silicon^16^. Consequently, the size of the primary silicon in Al–70 wt.%Si–1.0 wt.%P alloy is smaller than that in Al–70 wt.%Si alloy at the same undercooling. Meanwhile, the P addition decreased the critical undercooling for the morphological transition of primary silicon due to more nucleation sites. It also can be seen in Ge and its alloys with Sn and Si in a series of papers by Li and co-workers^17–19^.

### Microstructure

**Figure 4:**
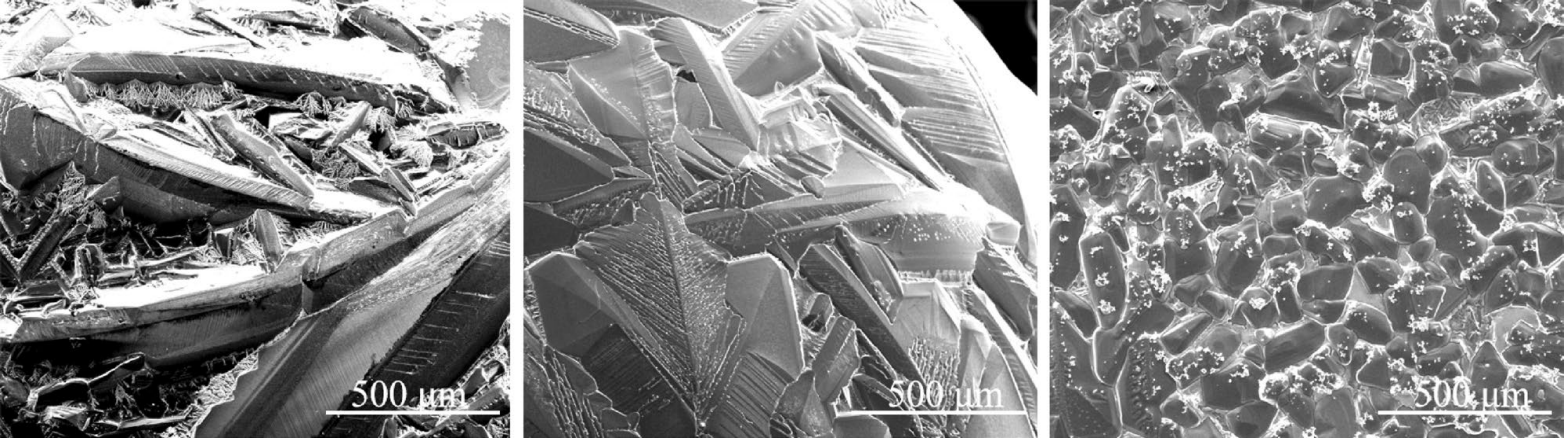
The SEM images of Al–70 wt.%Si alloys at different undercooling: (**a**) Δ*T* = 92 K, (**b**) Δ*T* = 154 K, (**c**) Δ*T* = 240 K.

**Figure 5:**
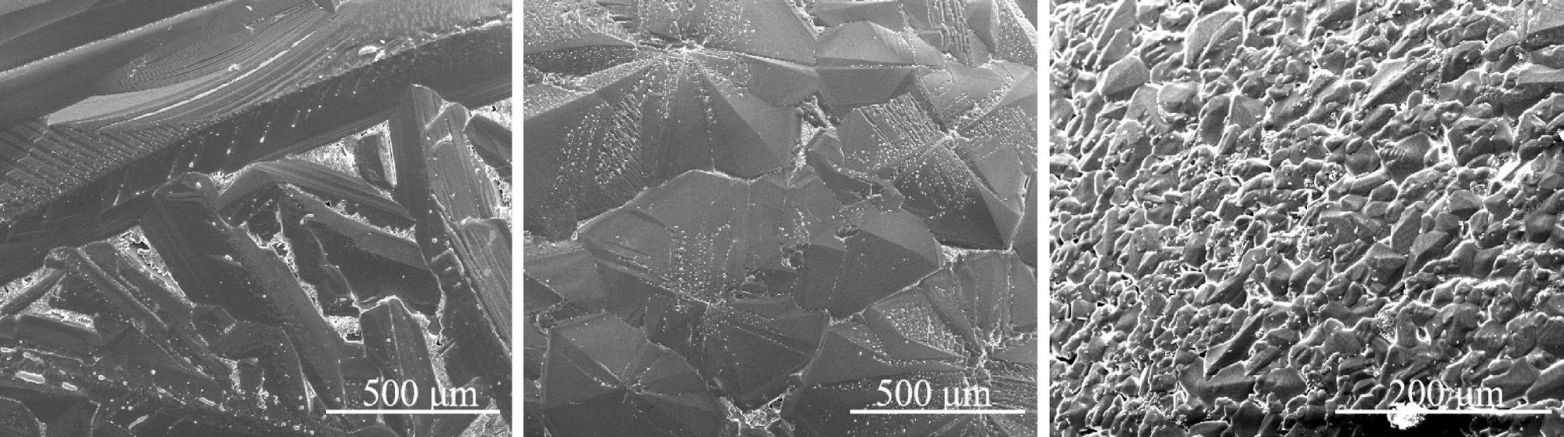
The SEM images of Al–70 wt.%Si–1.0 wt.%P alloys at different undercooling: (**a**) Δ*T* = 51 K, (**b**) Δ*T* = 141 K, (**c**) Δ*T* = 190 K.

The morphology of primary silicon observed by scanning electron microscopy at different undercooling shown in Fig. 4 for the Al–70 wt.%Si alloy and Fig. 5 for the Al–70 wt.%Si–1.0 wt.%P alloy. At low undercooling, a well-developed dendrite of primary silicon distributed randomly in the surface image. They are connected and forming a network. The blocky grains dominant the surface of the as-solidified sample at intermediate undercooling. The anisotropy of silicon plays a less important role in determining the morphology of crystal at intermediate undercooling than at low undercooling. The morphology of primary silicon is equiaxed and homogeneously distributed on the sample surface at large undercooling. Thus, the morphological change of the primary silicon indicates that the growth mode from lateral growth to non-lateral growth mode is not abrupt. It also can be seen in pure Si and pure Ge^6,14^.

The fact is that the morphology of primary silicon presents dendrites with several branches at low undercooling, block at intermediate undercooling, and equiaxed grains at large undercooling. No matter whether the alloy contains the P or not, the difference is only the critical undercooling of the morphological transition of primary silicon.

## Discussion

### Nucleation rate

**Figure 6:**
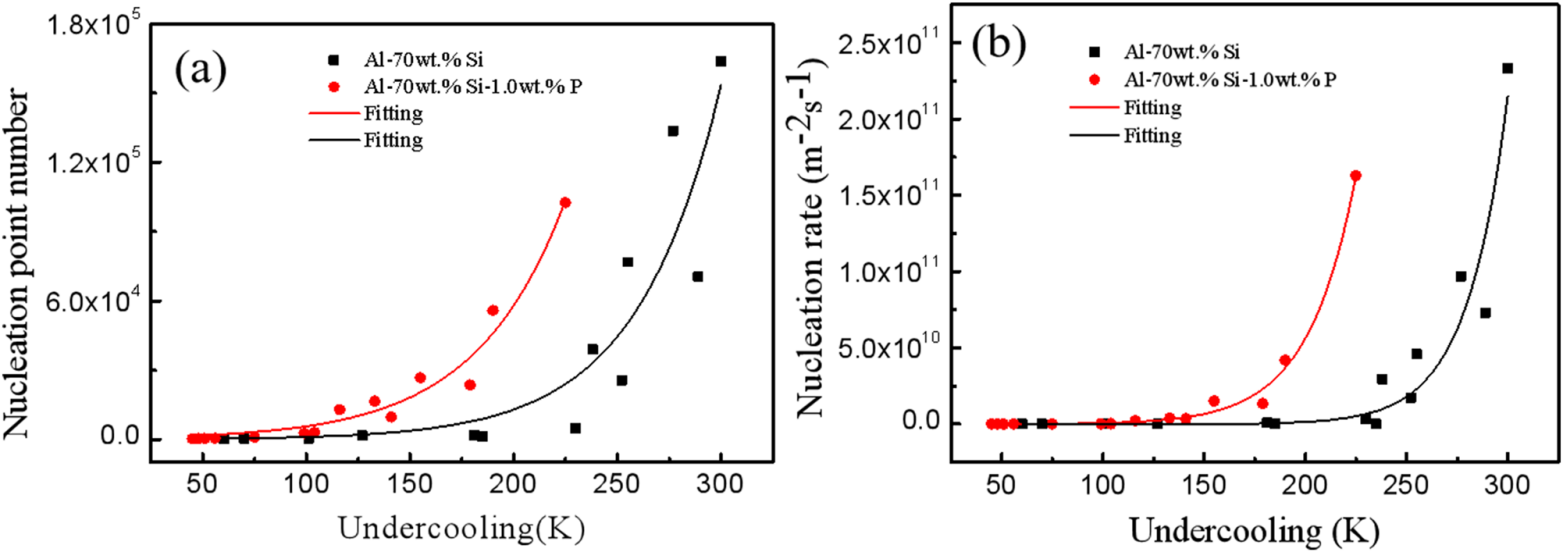
Measured nucleation data: (**a**) nucleation point number, (**b**) nucleation rate as a function of undercooling.

The nucleation rate is one of the key parameters that govern the grain size and distribution. The classical heterogeneous nucleation theory considered that the larger the nucleation rate, the more nuclei formed at the same time, and the finer the microstructure after solidification^20^. The common methods for measuring the nucleation rates for the Al–70 wt.%Si and Al–70 wt.%Si–1.0 wt.%P alloys are based on HSV images and SEM images. In Ref.^21^, the authors described and analyzed the advantages and disadvantages of the HSV and SEM methods in detail. In this paper, the nucleation numbers of the Al–70 wt.%Si and Al–70 wt.%Si–1.0 wt.%P alloys are calculated by the SEM method accurately. Then, the nucleation rate of the Al–70 wt.%Si and Al–70 wt.%Si–1.0 wt.%P alloys can be calculated, shown in Fig. 6.

The nucleation numbers for the two alloys can be fitted by *N* = exp(3.1832 + 0.0284Δ*T*) for the Al–70 wt.%Si alloy and *N* = exp(6.3394 + 0.0232Δ*T*) for the Al–70 wt.%Si–1.0 wt.%P alloy as shown in Fig. 6a. The nucleation rate can be fitted by *I* = exp(11.305 + 0.0495Δ*T*) for the Al–70 wt.%Si alloy and *I* = exp(16.276 + 0.0424Δ*T*) for the Al–70 wt.%Si–1.0 wt.%P alloy as shown in Fig. 6b. It is shown that the fitting relationship can describe the experimental results of the two alloys well.

### Growth velocities

Xu^21^ found that the growth velocity of each dendrite decreases with solidification time. We know that the solidification time of each sample decreases with undercooling, and undercooling plays an important role in the evolution of morphology of primary silicon. Thus, the relationship between growth velocity and undercooling is worth further study.

**Figure 7:**
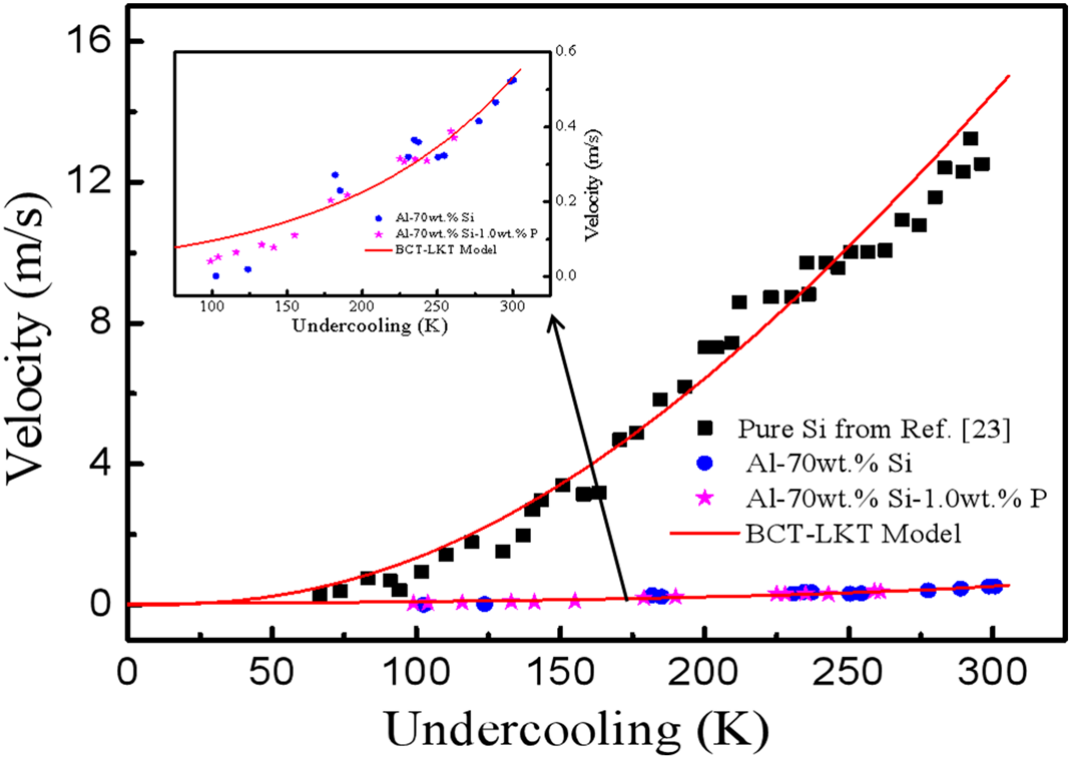
The measured growth velocity as a function of undercooling and model calculated result.

The experimentally measured growth velocity of primary silicon for the Al–70 wt.%Si and Al–70 wt.%Si–1.0 wt.%P alloys are shown in Fig. 7, and the growth velocity of pure Si is also shown for comparison. It can be found that all the growth velocities of silicon phases for pure Si and these alloys increase with undercooling. Meanwhile, the growth velocity of silicon phase in pure Si is far larger than that in the Al-70 wt.%Si and Al–70 wt.%Si–1.0 wt.%P alloys. Therefore, the composition (Al content and Si content) of the alloy plays a key role in the growth of the silicon phase. The P is added into Al–Si melt reduces the driving force required for the nucleation and growth of the silicon phase, decreases the critical undercooling for the morphological transition of the silicon phase, increases the nucleation number and nucleation rate of the silicon phase, and has a minor effect on the growth velocity of the silicon phase.

We also replotted predicted growth velocity for these alloys as calculated by BCT-LKT model as shown in Fig. 7. Accordingly, the total undercooling at the dendrite tip is assumed to be expressed as follows^22^:

2 $$ \Delta T_{p} = \Delta T_{c} + \Delta T_{t} + \Delta T_{r} + \Delta T_{k} $$

where Δ*T*_*c*_, Δ*T*_*t*_, Δ*T*_*r*_, Δ*T*_*k*_ is the solutal undercooling, the thermal undercooling, the curvature undercooling, the kinetic undercooling, respectively, and can be given as follows:

$$ \Delta T_{C} = m_{L} C_{0} \left[ {1 - \frac{{m^{\prime} /m_{L} }}{{1 - (1 - k)Iv(P_{C} )}}} \right] $$

$$ \Delta T_{t} = \frac{{\Delta H_{f} }}{{C_{P} }}Iv(P_{t} ) $$

$$ \Delta T_{r} = \frac{2\Gamma }{R} $$

$$ \Delta T_{k} = \frac{V}{\mu } $$

where *m*_L_ is the equilibrium liquidus slope, *m*′ is the kinetic liquidus slope, *C*_0_ is the composition of alloy, *k* is the kinetic solute partition coefficients, *I*_*v*_ is the Ivantsov function, *P*_*c*_ is the thermal Peclet numbers, Δ*H*_*f*_ is the heat of fusion, *C*_*p*_ is the specific heat of the liquid, *P*_*t*_ is the solution Peclet numbers, *Γ* is the ratio of the solid–liquid interface energy to the heat of fusion per unit volume, *R* is the radius of the dendrite tip, *V* is the growth speed of the dendrite tip, *μ* is the interfacial kinetic coefficient.

The material parameters used in the calculations are list in Table 2. As can be seen, the experimental data can be fitted well with the calculated by BCT-LKT dendrite growth model. The results will supply a foundation to confirm that the BCT-LKT model is suitable for Al–Si alloys.

**Table 2:** Material parameters used in calculations.

Parameter	Symbol	Unit	Value	References
Melting point	*T*_L_	K	1516	^13^
Specific heat of the liquid	*C*_p_	J/mol K	25.6	^23^
Thermal diffusivity of the liquid	*a*	m^2^/s	1.3 × 10^−5^	^23^
Solid–liquid interface energy	σ	J/m^2^	0.7271	^24^
Sound speed in the liquid	*V*_0_	m/s	3400	^23^
Diffusion coefficient	*D*_L_	m^2^/s	6.45 × 10^−9^	^25^
Equilibrium partition coefficient	*K*_*0*_		0.001554	Calculated
Diffusive speed	*V*_*D*_	m/s	7.9	Present
Entropy of fusion	Δ*S*	J/mol K	27.63	^24^
Slope of liquidus	*M*	K	0.02	^24^
Molar volume of Si	*V*_m_	m^3^/mol	12.96 × 10^−6^	^24^

## Conclusions

The morphology evolution of the primary silicon in Al–70 wt.%Si and Al–70 wt.%Si–1.0 wt.%P alloys was investigated by EML technique, in combination with in situ imaging of the solidification process by using HSV and microstructural analysis of the as-solidified samples by using SEM. The results show that:

- With increasing of undercooling, the morphologies of primary silicon in Al–70 wt.%Si and Al–70 wt.%Si–1.0 wt.%P alloys transformed from dendrites with several branches to blocky shape, and then to equiaxed grains, the morphology of growth interface changed from rough to smooth with the undercooling increasing from intermediate to high region, the refining element P decreased the critical undercooling for the morphological transition of the primary silicon.
- The nucleation number and nucleation rate increase exponentially with increasing of undercooling. The nucleation numbers can be fitted by *N* = exp(3.1832 + 0.0284Δ*T*) for the Al–70 wt.%Si alloy and *N* = exp(6.3394 + 0.0232Δ*T*) for the Al–70 wt.%Si–1.0 wt.%P alloy. The nucleation rate can be fitted by *I* = exp(11.305 + 0.0495Δ*T*) for the Al–70 wt.%Si alloy and *I* = exp(16.276 + 0.0424Δ*T*) for the Al–70 wt.%Si–1.0 wt.%P alloy.
- The average growth velocity of primary silicon measured in both materials as a function of undercooling could be fitted well by BCT-LKT model.

## Methods

The Al–70 wt.%Si and Al–70 wt.%Si–1.0 wt.%P alloys sample were prepared by arc melting the mixtures of silicon (99.9999 pct purity), aluminum (99.99 pct purity), and Cu-13%P master alloy in an argon atmophere. To ensure the compositional homogeneity, the alloys were remelted four times. Then the molten melts solidified and prepared with a diameter of 10 mm. Experiments to investigate the morphological transition of primary Si were using electromagnetic levitation (EML) method in a facility that can be evacuated and filled with gases (details in Refs.^14,26^). Firstly, the sample was placed on a sintered boron nitride holder positioned in the center of the EML coil. After the chamber of the EML facility was evacuated to 10^−4^ Pa using a turbomolecular pumpand then filled with purified argon gas, the sample was cyclically heated by a 200 W continuous wave CO_2_ laser, levitated by a radio-frequency generator. The sample was superheated to a temperature of 300 K above the liquidus temperatures for 300 s, then were cooled by switching off the continuous wave CO_2_ laser and then full with purified helium gas with different flow rate to achieve different undercooling. The temperature was measured by a monochromatic pyrometer with 2 ms response time and 1.55 μm operating wavelengths. A high-speed video that could reach 150,000 frames per second, and with the maximum resolution of 1280 × 1024 pixels, was used to capture the pictures during the solidification process to observe and record the growth morphology of primary silicon in the undercooled state. Morphologies of sample surfaces of the as-solidified samples were analyzed using scanning electron microscopy.

## References

[1] Lv GQ, Bao Y, Zhang YF, He YF, Ma WH, Lei Y (2018). Effects of electromagnetic directional solidification conditions on the separation of primary silicon from Al–Si alloy with high Si content. Mater. Sci. Semicond. Process..

[2] Li Y, Jiang T, Wei BW, Xu BY, Xu GM, Wang ZD (2020). Microcharacterization and mechanical performance of an Al–50Si alloy prepared using the sub-rapid solidification technique. Mater. Lett..

[3] Kang N, Coddet P, Ammar MR, Liao HL, Coddet C (2017). Characterization of the microstructure of a selective laser melting processed Al–50Si alloy: Effect of heat treatments. Mater. Charact..

[4] Li JH, Hage FS, Liu XF, Ramasse Q, Schumacher P (2016). Revealing heterogeneous nucleation of primary Si and eutectic Si by AlP in hypereutectic Al–Si alloys. Sci. Rep..

[5] He Y, Zhang HL, Li T, Wang XT (2013). Calculation of Jackson’s factor of Mg_2_Si in Mg melt using coordination polyhedron. J. Alloys Compd..

[6] Panofen C, Herlach DM (2007). Solidification of highly undercooled Si and Si–Ge melts. Mater. Sci. Eng. A.

[7] Wang J, Guo Z, Song JL, Hu WX, Li JC, Xiong SM (2018). On the growth mechanism of the primary silicon particle in a hypereutectic Al–20wt%Si alloy using synchrotron X-ray tomography. Mater. Des..

[8] Chen X, Zhong YB, Zheng TX, Shen Z, Wang J, Fan LJ, Zhai Y, Peng MH, Zhou BF, Ren WL, Lei ZS, Ren ZM, He Q (2017). Refinement of primary Si in the bulk solidified Al–20 wt.%Si alloy assisting by high static magnetic field and phosphorus addition. J. Alloys Compd..

[9] Gao T, Bian YH, Liu XF (2019). A novel Zn–Cu–P master alloy and its modification performance on primary Si of A390 alloy. Results Phys..

[10] Nie JF, Zhao YH, Li YS, Wang F, Yang HB, Hu KQ, Liu GL, Liu XF (2019). Reactive synthesis of hexagonal Ti_5_P_3.16_ crystals and their heterogenous nucleating mechanism on primary Si. J. Alloys Compd..

[11] Wang K, Lv X, Zhu YM, Jiang HY, Wang QD, Ye B, Ding WJ (2019). In-situ synthesis of novel Al–P–O master alloy and its refinement and modification effects on Si phases in hypereutectic Al–30Si alloys. Mater. Charact..

[12] Mendis BG (2019). Planck's generalised radiation law and its implications for cathodoluminescence spectra. Ultramicroscopy.

[13] Hernandez FCR, Djurdjevic MB, Kierkus WT, Sokolowski JH (2005). Calculation of the liquidus temperature for hypo and hypereutectic aluminum silicon alloys. Mater. Sci. Eng. A.

[14] Jian ZY, Nagashio K, Kuribayashi K (2002). Direct observation of the crystal-growth transition in undercooled silicon. Metall. Mater. Trans. A.

[15] Zhou XL, Wu YY, Li YF, Wu L, Gao T, Li H, Liu XF (2017). Absorbing formation mechanism of AlP on TiB_2_ substrate and their application as high-efficiency nucleating agent in Al–45Si alloy. J. Alloys Compd..

[16] Jiang T, Li SJ, Yu C, Fu JY, Wei BW, Luo LL, Xu GM (2019). The evolution on the microstructure and thermal expansion behavior of Al–50Si alloy with different P contents. J. Mater. Sci. Mater. Electron..

[17] Li DL, Volkmann T, Eckler K, Herlach DM (1995). Crystal growth in undercooled germanium. J. Cryst. Growth.

[18] Li D, Eckler K, Herlach DM (1996). Undercooling, crystal growth and grain structure of levitation melted pure Ge and Ge-Sn alloys. Acta Mater..

[19] Li DL, Eckler K, Herlach DM (1995). Evidence for transitions from lateral to continuous and to rapid growth in Ge–1at%Si solid solution. Europhys. Lett..

[20] Turnbull D (1952). Kinetics of solidification of supercooled liquid mercury droplets. J. Chem. Phys..

[21] Xu JF, Diao L, Yan JH, Dang B, Zhu M, Chang FE, Jian ZY (2016). In situ observations of the rapid solidification for undercooled Al_30_Si_70_ alloy melt. J. Mater. Res..

[22] Lipton J, Kurz W, Trivedi R (1987). Rapid dendrite growth in undercooled alloys. Acta Metall..

[23] Aoyama T, Takamura Y, Kuribayashi K (1999). Dendrite growth processes of silicon and germanium from highly undercooled melts. Metall. Mater. Trans. A.

[24] Jian ZY, Yang XQ, Chang FE, Jie WQ (2010). Solid-liquid interface energy between silicon crystal and silicon–aluminum melt. Metall. Mater. Trans. A.

[25] Zhang HW, Nakajima K, Wang EG, He JC (2012). Simulation of macrosegregation and solidification microstructure evolution for Al–Si alloy by coupled cellular automaton-finite volume model. Chin. J. Nonferrous Met..

[26] Dang B, Jian ZY, Xu JF, Yan JH (2017). Solidification of the undercooled Al–Si alloy containing 1.0 PctRE. Metall. Mater. Trans. A.

